# Supplementary material for: Asynchronous magnetic resonance elastography: Shear wave speed reconstruction using noise correlation of incoherent waves
Source: Magn Reson Med. 2022 Oct 27;89(3):990–1001. doi: 10.1002/mrm.29502 (PMC9792433; doi:10.1002/mrm.29502)
Supplement: Supplementary file 1 — DATA S1 MATLAB code used to generate the numerical simulations of Figure 1 [file MRM-89-990-s002.zip › k-Wave/helpfiles/example_na_modelling_nonlinearity.html]

Modelling Nonlinear Wave Propagation Example (k-Wave)


# Modelling Nonlinear Wave Propagation Example

This example describes the characteristics of the nonlinearity encapsulated by the first-order k-Wave simulation functions.

- Open the file in the MATLAB Editor
- Run the file in MATLAB

## Contents

- Cumulative nonlinearity
- Numerical errors

## Cumulative nonlinearity

The coupled governing equations used in the k-Wave simulation functions (`kspaceFirstOrder1D`, `kspaceFirstOrder2D`, and `kspaceFirstOrder3D`) are derived directly from the equations of fluid mechanics. The nonlinear terms are then re-written in terms of the acoustic Lagrangian density (which characterises the difference between the kinetic and potential energy density of the acoustic wave). If only cumulative nonlinear effects are important (these are nonlinear effects that "build-up" over space and time), the Lagrangian density can then be set to zero. This is sufficient for modelling nonlinear effects in many biomedical ultrasonics applications (in the absence of microbubbles). The strength of the nonlinear effects observed is dependent on the magnitude of the pressure field (the higher the pressure, the more nonlinear effects will be observed), the material parameter B/A (the parameter of nonlinearity), and the absorption parameters (the generation of higher frequency harmonics is delicately balanced with their absorption).

Within k-Wave, the parameter of nonlinearity is specified by `medium.BonA`. If this parameter is given, nonlinear governing equations are solved at each time step. If this is not given, linear governing equations are used (so the output results will always scale linearly with the input pressure). To illustrate the characteristics of the nonlinearity encapsulated by k-Wave, the time series recorded 15 wavelengths from the source for a shock parameter of 2 is compared with the series solution given by Mendousse (`mendousse`). There is a close agreement between the theoretical and numerical results which demonstrates that the desired nonlinear and absorption characteristics have been correctly modelled.


## Numerical errors

The accuracy of the k-Wave model is dependent on several parameters. First, the number of grid points used per wavelength will control whether the computational grid can support the propagation of the generated harmonics. In turn, the rate at which these harmonics are produced will depend on the shock parameter (for example, the source strength, the receiver distance, and the nonlinearity parameter), while the rate at which they are absorbed will depend on the power law absorption parameters. Finally, the CFL number will control the amount of unwanted numerical dispersion introduced by the finite-difference time step, as well as the accuracy with which the nonlinearity and absorption terms in the governing equations are computed. For strongly nonlinear fields, the number of points per wavelength at the fundamental frequency may need to be high to allow the generated harmonics to be modelled (giving at least two points per wavelength at the highest generated harmonic).
